# Global, regional, and national burden of type 2 diabetes mellitus caused by high BMI from 1990 to 2021, and forecasts to 2045: analysis from the global burden of disease study 2021

**DOI:** 10.3389/fpubh.2025.1515797

**Published:** 2025-01-23

**Authors:** Xin Huang, Yanyan Wu, Yulu Ni, Haiyan Xu, Yinhui He

**Affiliations:** Department of Endocrinology and Metabolism, The Fifth Affiliated Hospital of Wenzhou Medical University, Lishui Municipal Central Hospital, Lishui, China

**Keywords:** type 2 diabetes mellitus (T2DM), high body mass index (high BMI), deaths, disability-adjusted life years (DALYs), socio-demographic index (SDI), global burden of diseases (GBD)

## Abstract

**Objective:**

To produce estimates of the global burden of type 2 diabetes mellitus (T2DM) caused by high body mass index (high BMI) and its impact for 2021 and projections for 2045.

**Methods:**

We downloaded data from the Global Burden of Disease Study 2021(GBD 2021) to estimate the disease burden of T2DM caused by high BMI. Secondary analyses were performed by year, age, gender, region, and socio-demographic index (SDI).

**Results:**

Globally, the all-ages number of T2DM-related deaths has increased significantly from 238.1 thousand to 723.7 thousand, representing a 203.9% increase since 1990. And the all-ages number of T2DM-raleted DALYs has raised from 10.4 million to 39.3 million, increased by 276.7% from 1990. The burden was expected to continue to increase to 1296.7 thousand by 2045 for all-ages number of deaths, and 85.5 million by 2045 for all-ages number of DALYs. The curves of T2DM-related burden showed an intersection for different genders around the age of 60, beyond which women exhibit a higher burden, compared to men. The disease burden of T2DM caused by high BMI shows a significant upward trend across all SDI groups, with a heavier burden on women, especially in the postmenopausal female population. In 2021, among the 204 countries and territories, the top 3 largest number of T2DM-related burden caused by high BMI occurred in China, India, and United States. The top three countries with highest T2DM-related rate caused by high BMI were Fiji, Marshall Islands, and Kiribati.

**Conclusion:**

Our study reveals that the disease burden of T2DM caused by high BMI is significantly increasing and is expected to continue rising in the future. Women bear a heavier burden, particularly postmenopausal women, and there are significant differences in the disease burden across different geographical regions, and socioeconomic statuses. Targeted considerations and specific strategies are essential to address these disparities, thereby improving public health and reducing the burden.

## Introduction

Type 2 diabetes mellitus (T2DM) is a chronic condition characterized by the body’s inability to effectively use insulin, leading to high blood sugar levels. It is a significant public health concern worldwide, with a considerable impact on human life, quality of life, and health expenditures. T2DM is often associated with being overweight, physical inactivity, and genetics (1). It can lead to serious complications, including heart disease, stroke, kidney disease, nerve damage, and vision problems, making early diagnosis and management crucial (2). The prevalence of T2DM is on the rise, with an estimated 462 million individuals affected globally, corresponding to 6.28% of the world’s population. This represents a prevalence rate of 6,059 cases per 100,000 and is expected to increase to 7,079 individuals per 100,000 by 2030. The disease is the ninth leading cause of mortality, with over 1 million deaths attributed to diabetes annually (3–5). In terms of healthcare costs, the economic burden of T2DM is substantial. In the United States alone, the total direct and indirect estimated costs of diagnosed diabetes in 2022 were $413 billion, with total direct costs increasing from $227 billion in 2012 to $307 billion in 2022 (6). This highlights the importance of preventive measures and effective management strategies to reduce the disease burden and associated costs. Globally, the prevalence of T2DM shows a distribution pattern that matches socio-economic development, with developed regions such as Western Europe showing higher prevalence rates. There is also a concerning trend of rising prevalence in lower-income countries, indicating the need for urgent public health measures (7).

Obesity, defined by excessive body fat accumulation to an extent that it may impair health, is a global public health issue with significant consequences for both individuals and healthcare systems (8). The prevalence of obesity has been rising, with approximately 2.5 billion adults worldwide being overweight and over 890 million adults living with obesity in 2022 (9). This translates to 43% of adults aged 18 years and over being overweight, and 16% being obese, with variations observed across different regions and countries (10). The World Health Organization (WHO) uses the Body Mass Index (BMI) scale to categorize individuals as underweight, healthy, overweight, or obese, with a BMI of 25 to 30 indicating overweight and over 30 indicating obesity. The health consequences of obesity are substantial and include an increased risk of T2DM, heart disease, stroke, certain types of cancer, and other non-communicable diseases (NCDs) (11). In 2019, it was estimated that around 5 million deaths were attributable to obesity, highlighting its status as a leading risk factor for early death. The global distribution of health impacts from obesity shows that almost 10% of deaths in 2019 resulted from obesity-related consequences, with higher percentages observed in middle-income countries compared to high-income countries (7). The economic burden of obesity is also considerable. Medical costs associated with obesity include direct expenses such as hospitalizations, outpatient care, and prescription drugs, as well as indirect costs like reduced productivity and absenteeism. In the United States, the aggregate medical cost due to obesity was estimated at $260.6 billion in 2016, with significant variations in individual-level expenditures by state. Globally, if no action is taken, the costs of overweight and obesity are predicted to reach US$ 3 trillion per year by 2030 and more than US$ 18 trillion by 2060 (12).

In conclusion, T2DM is a growing epidemic with significant health, social, and economic implications. It is essential to address the modifiable risk factors through public health initiatives and improve the management of those affected to reduce the global burden of this disease. Efforts to address obesity involve a multifaceted approach, including public health initiatives, policy changes, and individual lifestyle modifications. Strategies such as promoting healthy diets, increasing physical activity, and creating supportive environments are crucial in combating obesity and its associated health and economic burdens. Therefore, this study aimed to comprehensively assess the burden of T2DM caused by high BMI by time, nation, age, gender, and socioeconomic status, and forecast toward 2045, expecting to provide essential information for targeted health policies.

## Methods

### Data collection

The Global Burden of Disease Collaborative Network provides comprehensive epidemiologic data on 288 causes of death, 371 diseases and injuries, and 88 risk factors across 204 countries and territories from 1990 to 2021. The global burden of diseases, injuries, and risk factors study (GBD) 2021 examines health trends worldwide. The study, which leverages 328,938 data sources, reveals health disparities across age, sex, location, and socioeconomic groups. Additionally, GBD 2021 examines the burden of non-communicable diseases and the contribution of risk factors such as high blood sugar, drug use, and obesity on health outcomes. Despite the challenges presented by current global threats, including antimicrobial resistance and climate change, the GBD 2021 study offers a cautiously optimistic outlook for the future of global health, advocating for evidence-based strategies to mitigate risks and enhance health outcomes. This data has been sourced from GBD 2021^1^ and is publicly accessible, eliminating the need for informed consent and ethics approval.

### Definition of high BMI

Risk factor means an attribute, behavior, exposure, or other factor which is causally associated with an increased (or decreased) probability of a disease or injury. If the probability decreased, the risk is a protective factor. High BMI was defined as BMI ≥ 25 kg/m2 for adults (aged 20+ years) and using thresholds from the International Obesity Task Force standards for children (aged <20 years). GBD 2021 incorporated data on high BMI from 246,703 data sources.^2^

### Definition of deaths, disability-adjusted life years and socio-demographic index

Deaths, disability-adjusted life years (DALYs), and socio-demographic index (SDI) are key concepts in the GBD 2021 study. Deaths refer to the deaths occurring in a population during a certain period. DALYs were the sum of years lost due to premature death (YLLs) and years lived with disability (YLDs). YLLs are calculated by subtracting the age at death from the longest possible life expectancy for a person at that age. YLDs are years lived with any short-term or long-term health loss. It is measured by taking the prevalence of the condition multiplied by the disability weight for that condition. Disability weights reflect the severity of different conditions and are developed through surveys of the general public. One DALY equals one lost year of healthy life. And the socio-demographic index (SDI) was applied as a measure that identifies where countries or other geographic areas sit on the spectrum of development. It is expressed on a scale of 0 to 1, with 0 being the lowest SDI value and 1 being the highest. SDI is based on three measures: (i) lag-distributed income per capita; (ii) average years of schooling in ages 15 and older; and (iii) total fertility rate (TFR) for females under age 25 (13).

### The auto-regressive integrated moving average model

ARIMA, which stands for Autoregressive Integrated Moving Average, is a statistical model used for time series forecasting. It combines three key components: (1) Autoregression (AR): This component captures the relationship between an observation and a certain number of lagged observations. It is based on the assumption that the value of a variable is a linear function of previous values; (2) Differencing (I): This is the process of stationarizing a non-stationary time series by removing the trend and seasonality components. Differencing involves subtracting the current observation from the previous one to achieve a stationary series; (3) Moving Average (MA): This component models the time series as a constant plus the average of a set number of error terms from previous observations. It captures the impact of random shocks or residuals on the current observation.

The ARIMA model is denoted as ARIMA (p, d, q), where p is the number of autoregressive terms, d is the number of times the raw observations have been differenced to achieve stationarity, and q is the size of the moving average window. The model is particularly useful for data that has a constant mean and variance over time, which is a characteristic of stationary data. It is widely used in economics, finance, and other fields where predictions about future values based on past observations are needed. However, it is important to note that ARIMA assumes that the underlying data generation process is linear and Gaussian, which may not always be the case in real-world scenarios.

### Statistical analysis

Data were presented as the value with a 95% uncertainty interval (UI). The age-standardized rates of YLDs were expressed as the number per 100,000 population. The Kruskal-Wallis test was used with nonnormal distributions to evaluate the difference in age-standardized rates between males and females. The autoregressive integrated moving average (ARIMA) model, widely used in time series analysis (6, 7), was applied to estimate the burden of vision loss attributable to diabetes from 2021 to 2045 (R system, version 4.2.2; detailed method in Supplementary materials). Most statistical analyses, except as specified above, were conducted using Prism software Version 9.0 (GraphPad, San Diego, California) and the Echarts open-source system. A *p* value less than 0.05 was considered statistically significant.

## Results

### Burden of T2DM caused by high BMI from 1990 to 2021

**Figure 1:**
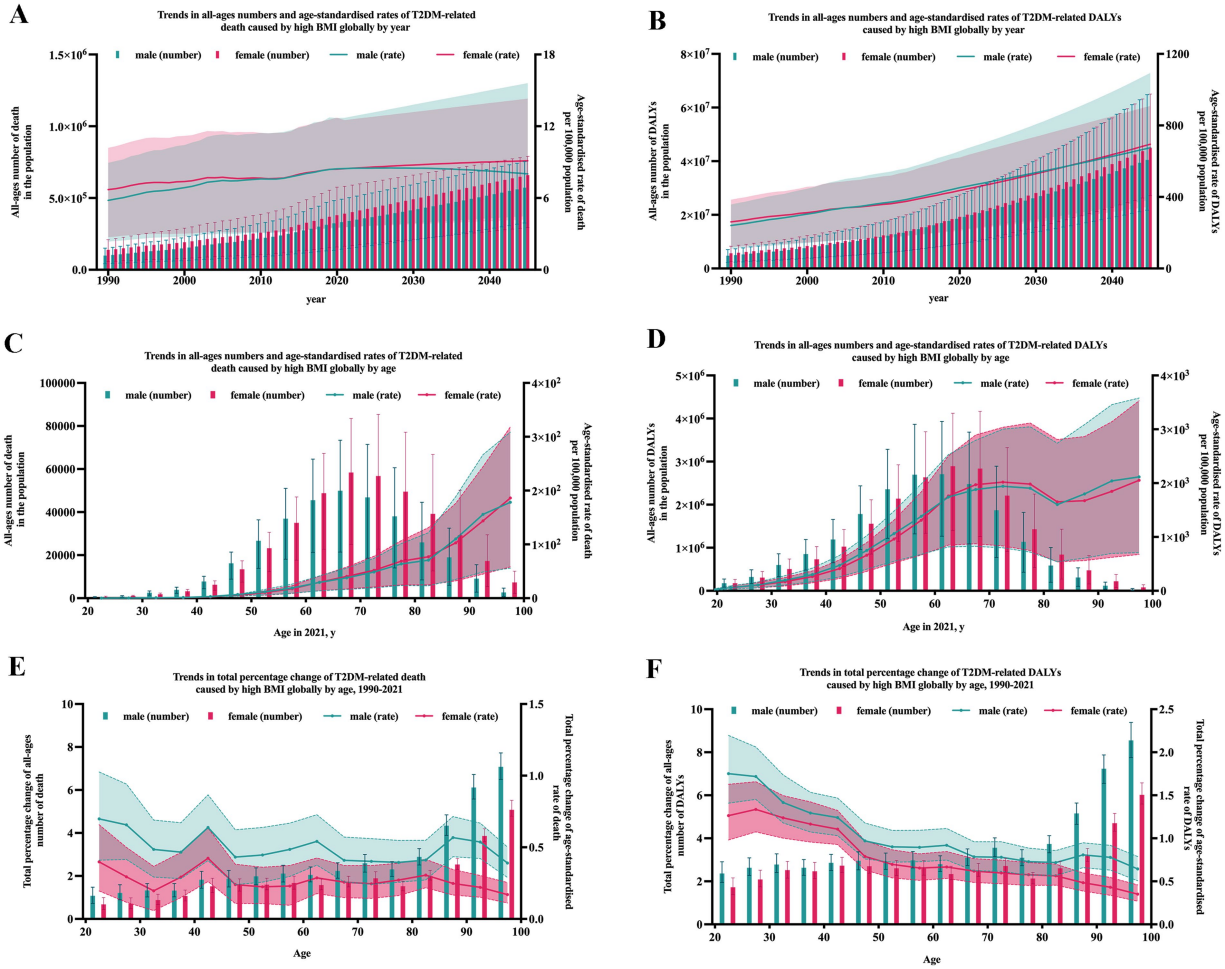
T2DM-related burden due to high BMI by year and age globally. Error bars indicate the 95% uncertainty interval (UI) for numbers. Shading indicates the 95% UI for rates.

The global burden of type 2 diabetes mellitus (T2DM) caused by high BMI has seen a significant increase from 1990 to 2021. Globally, the deaths of T2DM due to high BMI surged from 238.1 thousand (95% uncertainty interval [UI]: 98.9–358.3) in 1990 to 723.7 thousand (95% UI: 306.9–1075.4) in 2021, marking a 203.9.5% increase over the past 30 years. Meanwhile, the age-standardized death rate was 6.3 (95% UI: 2.5–9.6) per 100,000 population in 1990 and 8.5 (95% UI: 3.5–12.6) per 100,000 population in 2021. The number of DALYs in 1990 was 10.4 (95% UI: 4.9–315.3) million, increased to 39.3 (95% UI: 19.1–56.4) million, with an annual growth rate of 8.93%. The age-standardized rate of DALYs in 1990 was 251.5 (95% UI: 115.5–272.3) per 100,000 population and increased to 452.5 (95% UI, 220.4–650.5) in 2021, with an annual growth rate of 2.58% (Figures 1A,B).

### Burden of T2DM attributable to high BMI by age and sex

With age, both the global numbers of T2DM-related deaths and DALYs caused by high BMI exhibits a single-peaked curve, with peak occurring at age 70–74 (Figures 1C,D). While the age-standardized rates of T2DM-related deaths and DALYs caused by high BMI gradually increased with age (Figures 1C,D). As the T2DM burden due to high BMI also increased with time, to observe the growth rate in different age groups, percent change of burden was calculated to represent the annual rate of growth. The annual rate of growth of death and DALYs both peaked at age 40–44 (Figures 1E,F).

As shown in Figures 1A–F, the number of T2DM-related death and DALYs in females were constantly higher than that in males from 1990 to 2021. But for age groups, notably, an intersection in the curves of different genders around age 60, suggesting women bear a heavier burden of T2DM-related issues caused by high BMI compared to men after this age. For age-standardized rates of T2DM-realted deaths and DALYs, the trends stayed invariably, from 1990 to 2021, in all age groups. It is worth noting that with time and age, the difference between two genders grew obviously dramatic.

### Future prediction in the burden of T2DM caused by high BMI

Based on the trend observed, ARIMA model was applied to project the future trend to 2045. As estimated, as shown in Figures 1A,B, in 2045, there will be about 572.0 (95% UI: 325.9–753.2) thousand male deaths and 659.7 (95% UI: 294.1–790.1) thousand female deaths. And the death rate per 100,000 population will reach approximately 8.0 (95% UI: 4.5–15.6) in males and 9.1 (95% UI: 4.2–14.3) in females, respectively. And the DALYs rate will reach approximately 675.1 (95% UI: 384.6–1093.7) in males and 695.2 (95% UI: 347.5–909.2) in females.

### Burden of T2DM attributable to high BMI by countries and territories

**Figure 2:**
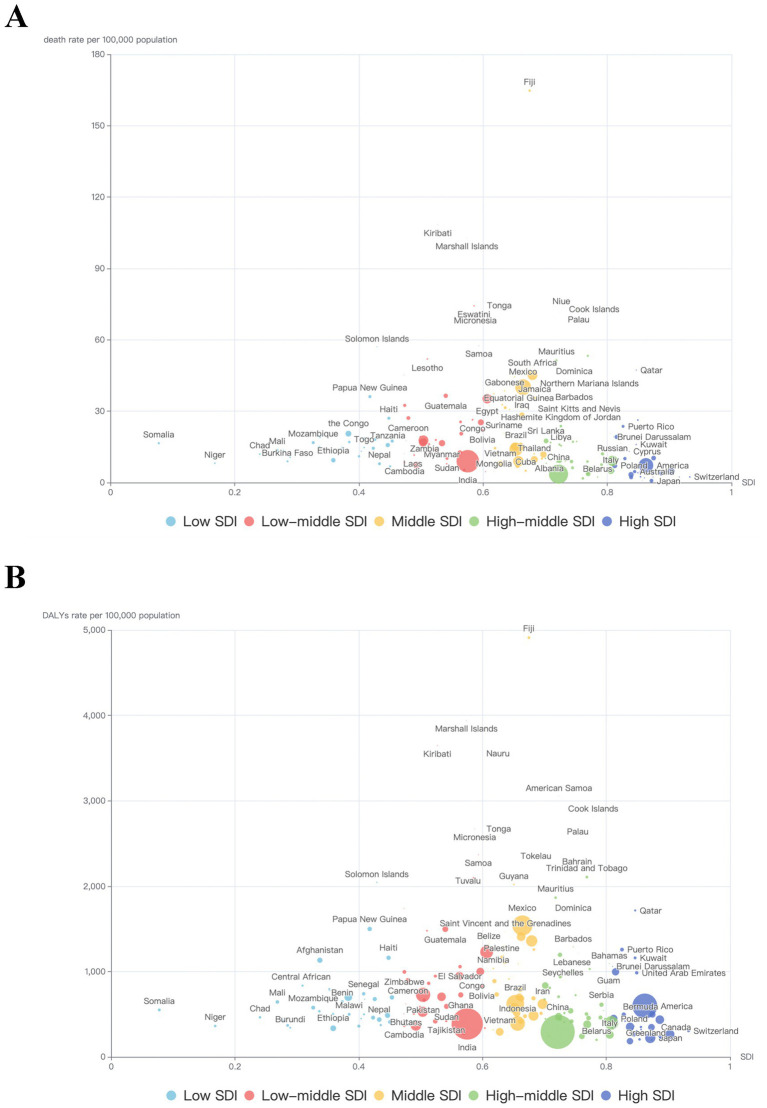
T2DM-related burden caused by high BMI for different SDI groups in 204 countries in 2021.

**Figure 3:**
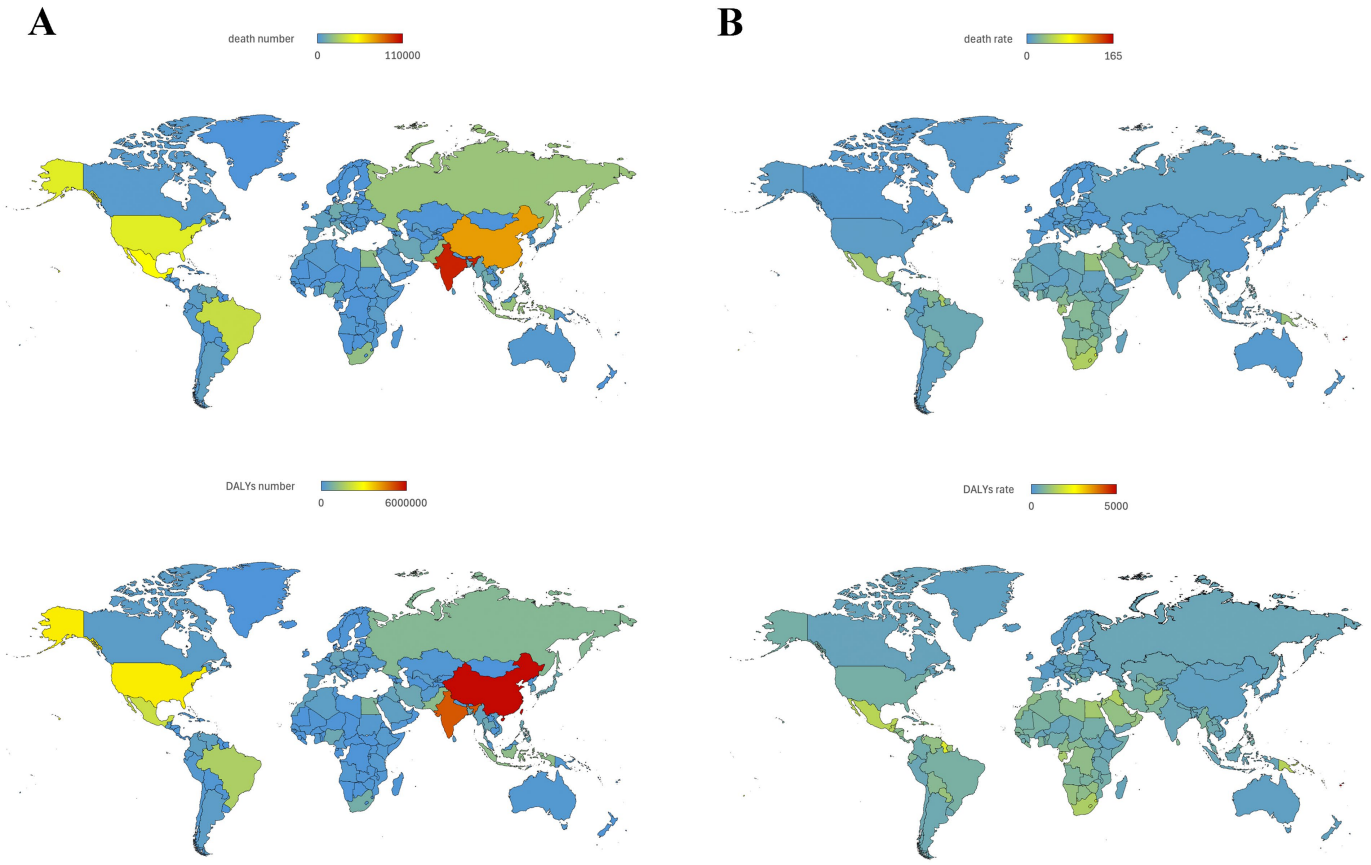
T2DM-related burden caused by high BMI in 204 countries in 2021.

In 2021, among the 204 countries and territories, the top three largest number of T2DM-related deaths caused by high BMI occurred in India: 102.0 thousand (95% UI: 36.5–161.0), China: 73.8 thousand (95% UI: 28.8–116.5), Mexico: 50.2 thousand (95% UI: 25.0–70.7). The top three countries with highest T2DM-related death rate caused by high BMI were Fiji: 164.7 (95% UI: 76.9–244.6), Kiribati: 108.3 (95% UI: 50.8–159.8), Marshall Islands: 102.8 (95% UI: 46.1–158.9). In contrast, Ukraine [1.78 (95% UI: 0.84–2.64)], Singapore [0.79 (95% UI: 0.30–1.24)] and Japan [0.77 (95% UI: 0.29–1.21)] had the lowest age-standardized death rate (per 100,000 population). As for DALYs, the highest numbers were observed in China [59.0 million (95% UI: 27.7–88.7)], India [49.6 million (95% UI: 20.3–75.2)], and United States [31.5 million (95% UI: 15.7–44.9)]. Fiji [4910.2 (95% UI: 2609.2–6943.0)], Marshall Islands [3940.1 (95% UI: 2048.1–5636.5)], Kiribati [3646.5 (95% UI: 1926.0–5120.0)] had the highest age-standardized rate (per 100,000 population). Ireland [199.6 (95% UI: 96.2–296.7)], Belarus [199.5 (95% UI: 101.0–286.1)] and French Republic [183.8 (95% UI: 87.4–274.7)] had the lowest age-standardized rate (per 100,000 population; Figures 2, 3).

### Burden of T2DM attributable to high BMI by SDI groups and health system grouping levels

**Figure 4:**
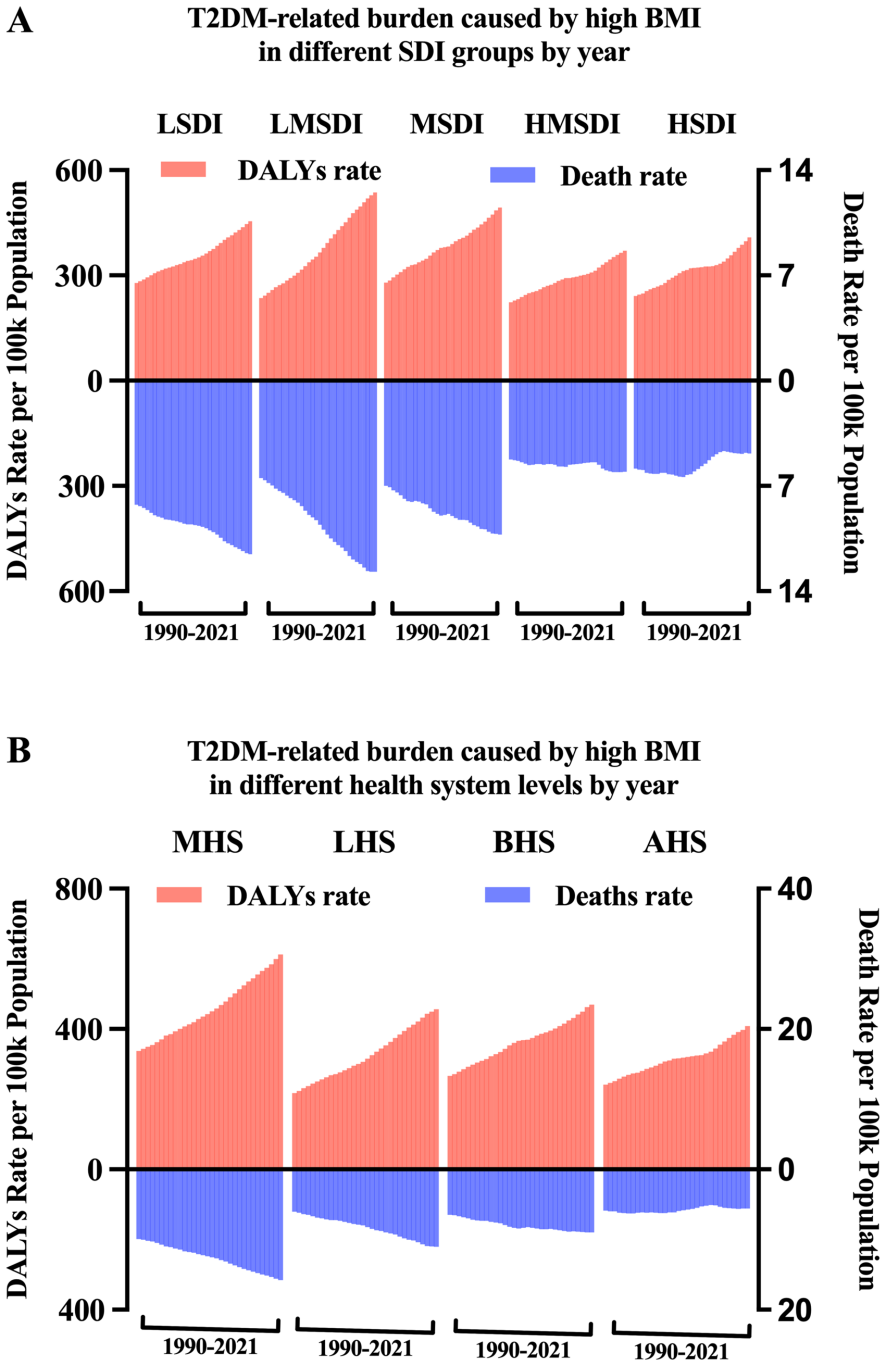
T2DM-related burden due to high BMI by year in different SDI groups and health system grouping levels.

Figure 4 showed the age-standardized death rate and DALYs rate per 100,000 population of T2DM due to high BMI by SDI groups and health system grouping levels. It seems there was an association between SDI and T2DM-related burden caused by high BMI, respectively. First, as shown in Figures 3, 4, in all SDI subgroups, both the death rate and DALYs rate had an evident trend of increasing over time, from 1990 to 2021. But from 1990 to 2021, highest death rates and DALYs rates always appeared in low-middle SDI regions. In GBD study 2021, it additionally provided information of health system grouping levels by location. Generally, the trend by health system grouping level was similar with that of SDI: from 1990 to 2021, the burden of HF increased in all levels of health system groups. The heaviest burden appeared in countries with minimal health system, and the lowest rates presented in countries with advanced health system (Figure 4B).

### Burden of T2DM attributable to high BMI by super regions

**Figure 5:**
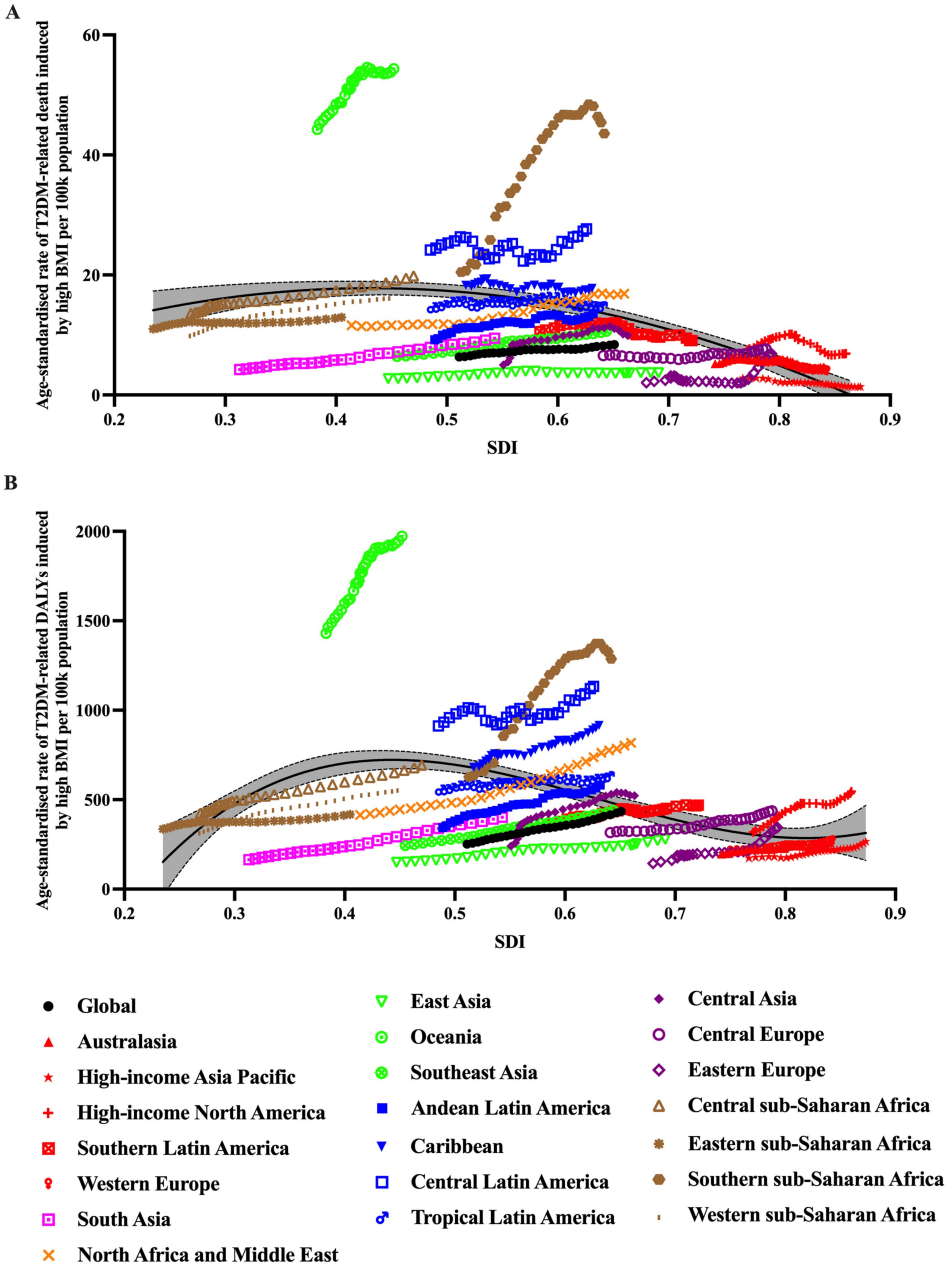
Trends in age-standardized rates of T2DM-related burden caused by high BMI for 21 GBD regions by SDI, 1990–2021.

In 2021, among the 21 super regions, the top three largest number of T2DM-related deaths caused by high BMI occurred in South Asia: 132.9 thousand (95% UI: 48.9–209.7), East Asia: 79.9 thousand (95% UI: 31.3–126.2), Southeast Asia: 70.6 thousand (95% UI: 28.1–110.3). The top 3 regions with highest T2DM-related death rate caused by high BMI were Oceania: 54.0 (95% UI: 24.1–79.8), Southern Sub-Saharan Africa: 43.9 (95% UI: 19.1–63.2), Central Latin America: 28.2(95% UI: 13.9–39.5). In contrast, High-income Asia Pacific [1.4 (95% UI: 0.5–2.2)], East Asia [3.7 (95% UI: 1.4–6.0)] and Western Europe [3.9 (95% UI: 1.5–5.9)] had the lowest age-standardized death rate (per 100,000 population). As for DALYs, the highest numbers were observed in South Asia [6.7 million (95% UI: 2.8–10.1)], North Africa and Middle East [4.3 million (95% UI: 2.3–5.8)], and East Asia [6.2 million (95% UI: 2.9–9.3)]. Oceania [1982.8 (95% UI: 980.2–2768.4)], Southern Sub-Saharan Africa [1308.0 (95% UI: 636.3–1799.1)], and Central Latin America [1179.3 (95% UI: 633.3–1599.4)] had the highest age-standardized rate (per 100,000 population). Australasia [268.5 (95% UI: 131.0–387.2)], Western Europe [281.6(95% UI: 138.3–409.7)] and High-income Asia Pacific [286.8 (95% UI: 128.7–443.1)] had the lowest age-standardized rate (per 100,000 population; Figure 5; Table 1).

**Table 1:** The number of death and DALYs, age-standardized rate of death and DALYs and its annual growth rate with the 95% uncertainty interval (UI; lower, upper) of T2DM-related burden caused by high BMI by genders, SDI groups, and GBD regions.

GBD 2021 super regions	T2DM-related death caused by high BMI						T2DM-related DALYs caused by high BMI					
	All-ages number		Age-standardized rate		Annual change of number (%)	Annual change of rate (%)	All-ages number		Age-standardized rate		Annual change of number (%)	Annual change of rate (%)
	(×1,000, 95%UI)		(/100,000, 95% UI)				(×1,000,000 95%UI)		(/100,000, 95% UI)			
	1990	2021	1990	2021			1990	2021	1990	2021		
Global	238.1(98.9,358.3)	723.7(306.9,1075.4)	6.3(2.5,9.6)	8.5(3.5,12.6)	6.58	1.09	10.4(4.9,15.3)	39.3(19.1,56.4)	251.5(115.5,372.3)	452.5(220.4,650.5)	8.93	2.58
Gender												
Male	99.3(42.3,150.8)	332.4(143.6,491.6)	5.8(2.4,9.0)	8.5(3.6,12.7)	7.58	1.47	4.8(2.2,7.0)	19.2(9.3,28.0)	240.0(109.3,357.9)	460.3(222.1,671.6)	9.77	2.96
Female	138.9(57.4,209.5)	391.3(163.8,581.0)	6.7(2.7,10.2)	8.4(3.6,12.5)	5.86	0.84	5.7(2.6,8.3)	20.1(9.8,28.7)	260.8(119.7,384.8)	444.6(218.2,634.1)	8.21	2.27
SDI grouping levels												
High SDI	64.9(26.1,97.0)	105.2(43.5,156.2)	5.9(2.4,8.7)	4.8(2.1,7.0)	2.00	−0.56	2.6(1.2,3.8)	7.3(3.5,10.6)	241.5(115.6,352.6)	408.8(205.1,583.6)	5.90	2.23
High-middle SDI	48.8(20.2,72.5)	119.4(49.9,178.3)	5.2(2.1,7.9)	6.1(2.5,9.1)	4.67	0.51	2.3(1.1,3.3)	7.0(3.5,10.2)	223.4(103.9,331.5)	370.6(186.1,536.2)	6.76	2.12
Middle SDI	67.9(29.0,101.4)	267.9(118.3,394.7)	7.0(2.8,10.7)	10.2(4.4,15.2)	9.49	1.49	3.2(1.5,4.7)	13.8(6.7,19.6)	280.0(127.2,417.5)	493.1(239.1,700.7)	10.58	2.46
Low-middle SDI	37.7(15.7,59.0)	174.3(73.0,261.4)	6.5(2.6,10.4)	12.7(5.1,19.6)	11.69	3.12	1.6(0.7,2.4)	8.4(3.9,12.3)	235.8(101.2,362.5)	536.8(242.0,788.2)	13.59	4.12
Low SDI	18.4(7.5,29.2)	56.0(23.3,87.1)	8.2(3.3,13.4)	11.5(4.4,18.5)	6.61	1.29	0.7(0.3,1.1)	2.7(1.2,4.0)	278.0(115.2,442.3)	454.4(195.0,684.4)	8.98	2.05
Health system grouping levels												
Advanced Health System	95.8(38.8,142.6)	167.3(69.4,244.8)	5.9(2.4,8.8)	5.6(2.5,8.0)	2.41	−0.15	3.9(1.8,5.7)	10.2(5.0,14.7)	241.6(115.0,350.3)	408.1(206.6,578.9)	5.30	2.22
Basic Health System	90.0(39.2,133.4)	329.3(149.3,480.3)	6.5(2.7,9.8)	9.0(3.9,13.3)	8.58	1.25	4.4(2.1,6.3)	18.1(9.2,25.6)	266.7(122.8,394.3)	469.5(236.9,666.2)	10.19	2.45
Limited Health System	46.0(18.4,73.8)	207.1(80.5,315.8)	6.0(2.3,10.0)	11.0(4.1,17.3)	11.29	2.66	2.0(0.8,3.1)	10.0(4.5,14.9)	217.3(88.1,346.2)	456.8(196.7,685.5)	13.21	3.56
Minimal Health System	5.9(2.4,9.4)	19.0(7.6,30.3)	9.9(4.0,16.2)	15.8(5.9,26.4)	7.19	1.89	0.2(0.1,0.4)	0.9(0.4,1.4)	338.0(143.4,536.9)	612.2(260.9,941.9)	9.95	2.62
High income												
High-income Asia Pacific	5.6(2.2,8.8)	6.9(2.4,11.6)	2.8(1.1,4.5)	1.4(0.5,2.2)	0.80	−1.60	0.4(0.2,0.5)	1.0(0.4,1.5)	173.5(78.7,265.4)	286.8(128.7,443.1)	5.42	2.11
Western Europe	36.9(13.8,56.9)	42.7(15.6,66.6)	6.1(2.3,9.4)	3.9(1.5,5.9)	0.52	−1.19	1.2(0.5,1.7)	2.2(1.0,3.2)	212.5(96.9,313.8)	281.6(138.3,409.7)	2.75	1.05
Australasia	1.2(0.5,1.8)	2.6(1.1,3.9)	5.2(2.1,7.8)	4.6(1.9,6.6)	3.84	−0.41	0.0(0.0,0.1)	0.1(0.1,0.2)	196.9(89.4,285.5)	268.5(131.0,387.2)	6.15	1.17
High-income North America	26.6(11.1,38.6)	45.6(20.4,63.5)	7.5(3.2,10.8)	6.9(3.2,9.5)	2.31	−0.26	1.1(0.5,1.5)	3.4(1.7,4.8)	318.0(158.7,446.1)	569.5(291.4,801.8)	7.10	2.55
Southern Latin America	4.8(2.1,6.9)	7.4(3.2,10.6)	10.6(4.5,15.4)	8.3(3.7,11.9)	1.76	−0.68	0.2(0.1,0.2)	0.4(0.2,0.6)	357.3(168.9,505.5)	466.8(233.0,658.3)	4.41	0.99
Central Europe, Eastern Europe and Central Asia												
Central Europe	9.8(4.3,14.1)	17.7(7.5,26.3)	6.5(2.8,9.5)	7.6(3.3,11.2)	2.59	0.52	0.5(0.2,0.7)	0.9(0.4,1.3)	316.4(149.0,452.7)	443.1(219.0,632.5)	2.96	1.29
Eastern Europe	5.9(2.6,8.3)	25.2(10.8,35.3)	2.1(0.9,2.9)	7.0(3.0,9.8)	10.63	7.68	0.4(0.2,0.6)	1.2(0.6,1.7)	143.8(69.8,205.2)	352.5(178.2,487.3)	6.34	4.68
Central Asia	2.4(1.1,3.3)	8.1(4.0,11.3)	5.0(2.2,7.0)	9.7(4.5,13.7)	7.82	3.05	0.1(0.1,0.2)	0.5(0.2,0.7)	240.8(120.4,338.1)	527.8(264.1,744.8)	9.69	3.85
Latin America and Caribbean												
Tropical Latin America	12.9(5.6,18.6)	36.6(15.8,52.5)	14.5(6.0,21.5)	14.4(6.2,20.9)	5.94	−0.03	0.6(0.3,0.8)	1.7(0.8,2.3)	553.2(260.7,790.2)	633.2(314.9,886.4)	6.45	0.47
Central Latin America	19.6(9.0,27.7)	70.3(35.1,97.6)	24.2(10.6,34.8)	28.2(13.9,39.5)	8.34	0.55	0.8(0.4,1.1)	3.1(1.7,4.1)	911.7(445.4,1269.4)	1179.3(633.3,1599.4)	8.55	0.95
Andean Latin America	1.9(0.8,2.7)	7.6(3.6,11.5)	9.2(3.9,13.8)	13.1(6.0,19.9)	10.03	1.35	0.1(0.0,0.1)	0.4(0.2,0.5)	343.8(164.6,490.4)	582.8(289.8,826.6)	11.86	2.24
Caribbean	4.6(2.0,6.8)	9.7(4.3,14.8)	18.3(7.7,27.2)	17.9(8.0,27.3)	3.53	−0.07	0.2(0.1,0.3)	0.5(0.2,0.7)	679.9(313.0,987.9)	938.4(449.5,1352.6)	5.68	1.23
Southeast Asia, East Asia, and Oceania												
East Asia	22.3(9.4,35.1)	79.9(31.3,126.2)	2.7(1.1,4.4)	3.7(1.4,6.0)	8.33	1.24	1.5(0.7,2.3)	6.2(2.9,9.3)	149.8(65.4,232.9)	296.2(138.8,443.3)	10.27	3.15
Southeast Asia	16.8(6.9,27.3)	70.6(28.1,110.3)	6.4(2.6,10.6)	10.8(4.1,17.3)	10.30	2.20	0.7(0.3,1.1)	3.3(1.5,5.0)	243.6(103.7,387.9)	467.1(199.4,714.0)	11.81	2.96
Oceania	1.3(0.6,2.0)	4.1(2.0,5.9)	44.2(19.6,69.7)	54.0(24.1,79.8)	6.69	0.71	0.1(0.0,0.1)	0.2(0.1,0.2)	1430.3(696.8,2148.9)	1982.8(980.2,2768.4)	8.29	1.25
North Africa and Middle East												
North Africa and Middle East	17.7(7.9,25.7)	70.4(34.8,98.7)	11.6(4.8,17.2)	17.2(8.0,24.4)	9.62	1.55	0.8(0.4,1.0)	4.3(2.3,5.8)	414.8(194.6,589.6)	854.4(431.8,1180.2)	15.12	3.42
South Asia												
South Asia	23.2(8.7,38.2)	132.9(48.9,209.7)	4.2(1.5,7.2)	9.5(3.4,15.3)	15.29	4.01	1.1(0.4,1.7)	6.7(2.8,10.1)	164.4(62.9,268.3)	413.8(172.4,626.0)	16.46	4.89
Sub-Saharan Africa												
Southern Sub-Saharan Africa	5.3(2.2,7.8)	23.4(10.6,32.8)	20.5(8.4,30.8)	43.9(19.1,63.2)	11.03	3.69	0.2(0.1,0.3)	0.8(0.4,1.1)	626.4(286.6,888.4)	1308.0(636.3,1799.1)	10.84	3.51
Eastern Sub-Saharan Africa	8.2(3.4,12.9)	21.6(9.0,34.7)	11.0(4.3,17.6)	13.3(5.2,22.2)	5.27	0.68	0.3(0.1,0.4)	0.9(0.4,1.3)	336.5(137.5,531.5)	433.8(181.1,669.3)	6.42	0.93
Central Sub-Saharan Africa	3.0(1.2,4.8)	10.7(4.2,17.1)	13.6(5.4,22.2)	20.7(7.7,34.4)	8.35	1.69	0.1(0.0,0.2)	0.5(0.2,0.7)	425.1(178.7,689.2)	733.0(304.1,1127.0)	10.96	2.34
Western Sub-Saharan Africa	8.4(3.2,13.3)	29.7(11.7,45.3)	10.2(3.8,16.6)	16.8(6.2,26.4)	8.21	2.09	0.3(0.1,0.5)	1.3(0.6,1.9)	324.6(133.2,514.1)	581.7(256.5,873.9)	10.38	2.55

## Discussion

This study presented a time trend of the burden of type 2 diabetes mellitus (T2DM) caused by high BMI from 1990 to 2021, and its global distribution by age, gender, nation and socioeconomic levels. This allowed us to gage the impact of aging, significant regional disparities, and gender-based differences on the burden of the disease. Our findings revealed a significant upward trend in the age-standardized rates of T2DM-related burden caused by high BMI, coupled with a sustained increase in the overall numbers of high BMI induced T2DM burden across all age groups. The study also highlighted considerable variations in the trend and magnitude of high BMI induced T2DM burden, evident in diverse age groups, geographical and socioeconomic regions, as well as between genders.

Though the disease burden maintained a consistent upward trend globally, differences in geographical distribution is also observed. At the GBD regional level, Oceania bear the heaviest burden. At the national level, Fiji, Kiribati, and Marshall Islands, bear the highest age-standardized burden rate. These countries were accompanied with low-middle or middle economic level, and minimal or limited health system. These regions and countries with heavier burden should be given priority in the future healthcare policy.

Obesity is a major risk factor for the development of T2DM, and the two conditions often coexist, exacerbating health complications and increasing healthcare costs. The escalating disease burden of T2DM attributed to obesity can be attributed to a multitude of factors, which have been outlined in various studies. First, the global rise in obesity rates is a significant factor contributing to the increased burden of T2DM (5). Second, unhealthy diets and a lack of physical activity are leading causes of obesity. The consumption of high-calorie, high-fat foods, coupled with a sedentary lifestyle, promotes weight gain and increases the risk of developing T2DM (14). Third, with economic development and urbanization, there is a shift toward diets high in processed and fast foods, which are often energy-dense and nutrient-poor (15). This, along with reduced physical activity due to more sedentary jobs and modes of transportation, contributes to obesity and related health issues. Moreover, aging populations are at higher risk of developing T2DM. As life expectancy increases and populations age, the prevalence of T2DM is expected to rise. Environmental factors, including chemical exposures and changes in the microbiome, have been implicated in the development of obesity and metabolic disorders, including T2DM. Inadequate access to healthcare and lack of effective diabetes management strategies can lead to uncontrolled diabetes and increased complications, thereby increasing the disease burden. The increasing disease burden of T2DM due to obesity underscores the need for comprehensive public health strategies that address these factors through prevention, early diagnosis, and effective management of both obesity and diabetes.

Besides, we found the burden was higher in countries with low-middle or middle SDI, and minimal health system. The possible reasons behind can be complex and multifaceted. Firstly, many low- and middle-income countries are undergoing rapid epidemiological transitions, with a shift from infectious diseases to non-communicable diseases like T2DM, often exacerbated by obesity. Secondly, as countries experience economic development and urbanization, there is a shift toward more sedentary lifestyles and diets high in processed and high-calorie foods, leading to increased obesity and subsequently, T2DM (16). Third, in low to middle SDI countries, the healthcare infrastructure may not be equipped to manage the increasing number of diabetes cases effectively. This includes limited access to diabetes medications, technologies, and healthcare professionals, which can result in higher morbidity and mortality rates (3). Moreover, the economic burden of diabetes, including direct medical costs and indirect costs due to lost productivity, can be particularly challenging for low to middle SDI countries with less resources to allocate to healthcare (4). Many low to middle SDI countries are experiencing population growth and aging, which are associated with higher prevalence of chronic conditions like T2DM (17). To mitigate this burden, comprehensive strategies are needed, including improving access to healthcare, promoting healthy lifestyles, and implementing policies that address the social and environmental factors contributing to obesity and diabetes. Additionally, international support and collaboration can help these countries strengthen their healthcare systems and implement effective diabetes management programs.

Gender disparity in the burden was detected. Though in all age groups, globally, the burden of both gendered increased from 1990 to 2021. However, female consistently bear a higher rate of burden, especially after 55 years of age. Previous studies also found similar patterns of gender disparity. The possible reasons behind can be complex and multifaceted. Biologically, women tend to have a higher percentage of body fat than men, even at the same BMI, which can lead to a higher risk of insulin resistance and T2DM (18). Additionally, hormonal changes, such as those occurring after menopause, can influence insulin sensitivity and glucose metabolism, contributing to a higher risk of T2DM in women. Sociocultural factors include differences in lifestyle, dietary habits, and physical activity levels between genders (19). Women may have different dietary patterns, such as lower consumption of certain protective foods like pulses or beans, and higher consumption of high-risk foods like eggs, chicken, or meat. Additionally, women’s roles and responsibilities, including childcare and household duties, can limit opportunities for physical activity, further contributing to obesity and diabetes risk (20). Women may have different patterns of healthcare utilization, including more frequent visits to healthcare providers. While this could potentially lead to earlier diagnosis and treatment, it may also result in a higher recorded prevalence of obesity and related conditions like T2DM (21). To address these disparities and reduce the disease burden of T2DM in women, a multifaceted approach is necessary. This includes promoting healthy lifestyles, improving access to healthcare, and developing sex-specific treatment strategies that consider the biological and sociocultural factors that contribute to the higher disease burden in women. Further research is needed to better understand the mechanisms underlying these sex differences and to inform more effective prevention and treatment strategies.

This supplementary analysis of the GBD data effectively addresses the previously uncharted territory of the impact of high BMI on the burden of T2DM at a global and national scale, differentiated by time, age, sex, SDI, and other factors. The study utilized established methodologies and incorporated the most recent data to forecast future trends. However, it is imperative to acknowledge several limitations. Firstly, the GBD’s data source and approach have certain weaknesses, primarily due to the variable quality of the data and a scarcity of population-based studies in less affluent areas. Secondly, disparities in health information systems naturally lead to some gaps in the data. Thirdly, variations in health information systems across different nations and regions also result in discrepancies. For instance, Asians typically have a higher body fat percentage compared to Caucasians of similar age, sex, and BMI, which suggests that employing uniform cut-off points could potentially lead to an underestimation of the associated risks among Asian populations.

## Conclusion

**Figure 6:**
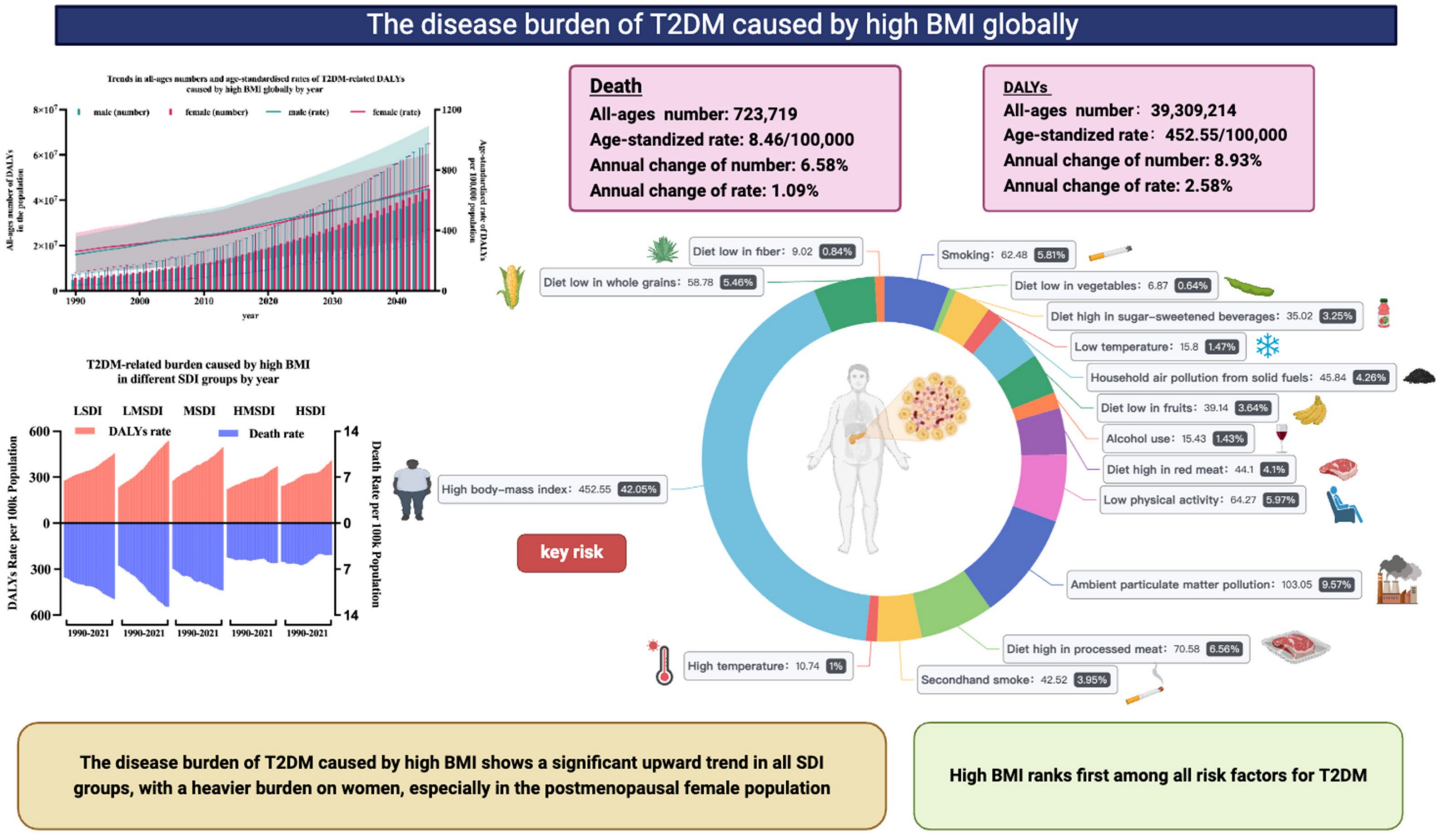
The main findings of the study.

To sum up, the research presented a comprehensive overview of the worldwide health impact of T2DM linked to elevated BMI levels, spanning from 1990 to 2021, and categorized by country, gender, age group, SDI, and geographical region. The findings indicate that, with the passage of time, and looking ahead to the year 2045, T2DM resulting from high BMI will persist as a significant public health issue. There is an urgent need for healthcare initiatives that prioritize metabolic health and for an increased focus on the prevention and management of T2DM and obesity. It is anticipated that the insights from this study will inform and guide strategic policy decisions in the healthcare sector (Figure 6).

## Data Availability

The original contributions presented in the study are included in the article/Supplementary material, further inquiries can be directed to the corresponding authors.

## Footnotes

^1^ https://vizhub.healthdata.org/gbd-results/

^2^ http://ghdx.healthdata.org/gbd2021/data-input-sources
